# Optical performance of monocrystalline and polycrystalline ceramic brackets: a prospective in vivo study of color stability and light transmittance

**DOI:** 10.2340/biid.v13.46474

**Published:** 2026-07-23

**Authors:** Elif Nadide Akay Polat, Berza Yilmaz

**Affiliations:** a Department of Orthodontics, Faculty of Dentistry, İstanbul Medeniyet University, Istanbul, Turkey; b Department of Orthodontics, Faculty of Dentistry, Bezmialem Vakıf University, Istanbul, Turkey

**Keywords:** Aesthetic bracket, ceramic bracket, color stability, light transmittance, radiometer, spectrophotometer

## Abstract

**Background/Objective:**

Ceramic brackets are widely used in aesthetic orthodontics; however, their optical stability under intraoral conditions remains incompletely understood. While previous studies have predominantly assessed color change in vitro, prospective clinical data simultaneously evaluating both chromatic parameters and light transmittance are scarce. This study aimed to evaluate the in vivo color stability and light transmittance of monocrystalline and polycrystalline ceramic brackets after 3 months of intraoral exposure, and to determine whether crystalline structure influences the magnitude of optical degradation.

**Materials and Methods:**

Fifty patients (13–22 years) were included in this prospective split-mouth longitudinal clinical study. Eight ceramic bracket types were evaluated: four monocrystalline (3M™ Gemini Clear, Inspire Ice™, Hubit™ Perfect Clear II, AO™ Radiance Plus™) and four polycrystalline (3M™ Clarity™ Advanced, AO™ 20/40, Dentsply Sirona Resolve™, GC™ Chic). Brackets were ligated onto archwires without bonding to enamel to prevent composite-induced discoloration. Color measurements (CIE L*a*b*) were obtained using a VITA Easyshade Compact spectrophotometer, and light transmittance was measured with an LEDex CM4000 radiometer at baseline (T0) and after 3 months (T1). Nonparametric tests (Mann–Whitney U, Kruskal–Wallis, and Wilcoxon signed-rank) were used for statistical analysis (*p* < 0.05).

**Results:**

All brackets exhibited statistically significant color and transmittance changes after intraoral exposure (*p* < 0.001). All brands demonstrated ‘extremely marked’ color changes according to National Bureau of Standards (NBS) criteria (NBS > 6). 3M™ Clarity™ Advanced showed the highest ΔE (median: 11.22 [interquartile range, IQR: 8.48–13.31]), whereas AO™ Radiance Plus™ exhibited the greatest transmittance reduction (ΔTr: median −10.00 [IQR: −15.00 to −5.00]). A weak negative correlation was observed between ΔE and ΔTr. Crystalline structure significantly influenced baseline optical parameters (Tr0, L0, a0) but did not determine the overall magnitude of color change (ΔE) or transmittance loss (ΔTr).

**Conclusions:**

Intraoral exposure significantly impairs the optical properties of aesthetic ceramic brackets. Crystalline structure influences baseline optical properties and the direction of chromatic shift (Δa, medium effect; Δb, small effect) but not the overall magnitude of color change (ΔE) or transmittance loss (ΔTr). Brand-specific manufacturing characteristics appear to be the primary determinants of long-term aesthetic performance.

**Key messages**

1. All evaluated ceramic bracket brands demonstrated clinically significant and ‘extremely marked’ color changes (National Bureau of Standards [NBS] > 6) and reduced light transmittance after 3 months of intraoral exposure, regardless of crystalline structure.
2. Crystalline structure (monocrystalline vs. polycrystalline) influenced baseline optical properties and the direction of chromatic shift but did not significantly determine the overall magnitude of color change or transmittance loss.
3. Brand-specific manufacturing processes and surface characteristics appear to be the primary determinants of optical stability, highlighting the importance of brand selection in clinical practice.

## Introduction

Increasing aesthetic expectations among adult orthodontic patients have led to the development of appliances that offer both clinical efficiency and improved aesthetics. Stainless-steel brackets remain the gold standard for their mechanical strength and fracture resistance. However, their metallic appearance can reduce patient acceptance [1, 2]. This limitation led to early polymer-based aesthetic brackets, such as polycarbonate systems. Despite their improved appearance, these showed clinical limitations, including slot deformation, creep, and poor color stability in the oral environment [3].

To overcome the shortcomings of early aesthetic brackets, ceramic brackets were introduced in the late 1980s. Contemporary ceramic brackets are predominantly fabricated from high-purity alumina (Al₂O₃) and are mainly available in monocrystalline and polycrystalline forms. Monocrystalline brackets are produced by controlled solidification of molten alumina, forming a single-crystal structure without grain boundaries, resulting in superior optical homogeneity and translucency [4]. In contrast, polycrystalline brackets are manufactured by sintering multiple alumina grains, generating crystal interfaces that increase light scattering and may influence optical transmission. Although reinforced and hybrid ceramic designs have since been developed [5], monocrystalline and polycrystalline alumina brackets remain the standard reference materials for evaluating intrinsic optical properties.

Aesthetic stability is critical for ceramic brackets because they are susceptible to clinical discoloration [6]. Both extrinsic staining from dietary chromogens and surface wear, as well as intrinsic alterations from ultraviolet exposure, thermal fluctuations, and biofilm accumulation, affect optical quality [7–10].

To evaluate color alterations, the CIE L*a*b* system is used. It represents color in three dimensions: lightness (L*, 0–100), the green-red axis (a*), and the blue-yellow axis (b*). Overall color difference (ΔE*) indicates the magnitude of chromatic change between measurements. A color difference of 1.0 is perceptible under controlled conditions, while values above 3.7 represent the threshold for clinical detectability in dentistry [4, 11].

Optical transmittance – the proportion of incident light passing through a material – is determined by microstructure and surface characteristics [12]. Monocrystalline brackets, which lack grain boundaries, typically exhibit higher transmittance, but variations in geometry and manufacturing processes can lead to brand-specific differences [13, 14]. Light transmittance is relevant for both aesthetics and light-cured adhesive polymerization, yet little research has addressed its in vivo stability [14]. Previous studies [15–17] highlight the need for in vivo research to clarify how chromatic changes interact with the oral environment.

Despite growing interest in aesthetic bracket performance [1, 7, 15–17], a critical gap remains in the literature: prospective clinical data simultaneously evaluating both chromatic parameters and optical transmittance under intraoral conditions are scarce [18]. Most available evidence derives from in vitro studies using artificial aging protocols, which may not adequately replicate the complexity of the oral environment, including salivary enzymatic activity, pH fluctuations, thermal cycling, and biofilm formation. Furthermore, whether crystalline structure (monocrystalline vs. polycrystalline) determines the magnitude of optical degradation – rather than merely influencing baseline optical properties – has not been directly addressed in a prospective clinical setting. The present study was specifically designed to fill this gap by simultaneously assessing color stability and light transmittance under standardized in vivo conditions across eight commercially available ceramic bracket brands.

Therefore, the present study aimed to evaluate the extent to which ceramic brackets maintain color stability and light transmittance after short-term intraoral exposure and to compare these optical parameters between monocrystalline and polycrystalline systems. The null hypothesis posited that short-term intraoral exposure would not produce significant changes in color stability or light transmittance, and that there would be no difference between monocrystalline and polycrystalline brackets.

## Materials and methods

### Study design and ethical approval

This prospective clinical study was approved by the Bezmialem Vakıf University Interventional Ethics Committee (Approval No: 71306642-050.01.04) and conducted in accordance with the Declaration of Helsinki. Informed consent was obtained from all participants (or their legal guardians for minors) prior to enrollment.

The study followed a longitudinal split-mouth sequential crossover design including both in vivo and in vitro components. In this design, each participant served as their own control by receiving both monocrystalline and polycrystalline bracket sets in two consecutive 3-month phases. The allocation sequence for the first phase (monocrystalline vs. polycrystalline) was determined by simple randomization using a computer-generated random number list, with allocation concealed in sealed opaque envelopes opened at the time of bracket placement. To minimize potential carry-over effects, a 2-week washout period was implemented between phases, during which no brackets were worn. Additionally, the split-mouth design ensured that both bracket types were evaluated within the same oral environment, controlling for inter-individual variability in dietary habits, salivary composition, and oral hygiene.

### Sample size determination

An a priori power analysis, based on previous investigations [18, 19] and calculated using the ΔE parameter (α = 0.05, power = 0.80), indicated a minimum requirement of 45 brackets per brand. To account for potential dropouts, 50 patients were enrolled. Each participant sequentially received both monocrystalline and polycrystalline bracket sets over two consecutive 3-month phases, yielding 50 clinical samples per brand (400 clinical brackets in total). Additionally, five control brackets per brand (40 brackets in total) were stored in artificial saliva as an in vitro reference [16].

### Eligibility criteria

Inclusion criteria comprised non-smoking individuals aged 13–22 years with permanent dentition, good general health, and excellent oral hygiene (Plaque Index ≤ 1). Patients were enrolled after completion of the leveling and alignment phase to standardize brushing conditions and minimize plaque-retentive variability. Exclusion criteria included systemic diseases affecting salivary composition, use of medications that influence salivary flow, active periodontal disease, and high consumption of chromogenic agents.

### Dietary standardization

To minimize intra-group variability related to dietary habits, all participants received standardized oral hygiene instructions at enrollment and at each follow-up visit. Patients were advised to limit consumption of strongly chromogenic beverages (e.g. coffee, tea, red wine, cola) to no more than one serving per day and to rinse with water immediately after consumption. Compliance was monitored through structured dietary recall questionnaires administered at each visit. Although dietary intake was not objectively controlled beyond these measures, this standardization protocol was applied consistently across all participants to reduce potential confounding bias.

### Bracket selection and allocation

**Figure 1:**
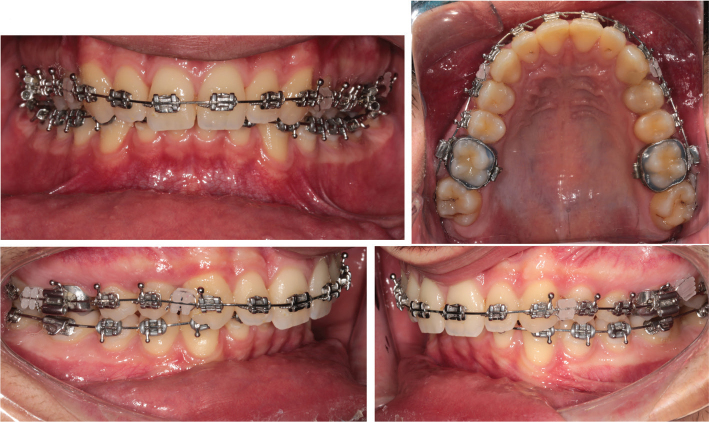
The brackets ligated on the arch wire between upper molars and between upper canines and first premolars.

Eight ceramic bracket systems were evaluated: four monocrystalline (3M™ Gemini Clear, Inspire Ice™, Hubit™ Perfect Clear II, AO™ Radiance Plus™) and four polycrystalline (3M™ Clarity™ Advanced, AO™ 20/40, Dentsply Sirona Resolve™, GC™ Chic) (Table 1). Within each 3-month phase, brackets were distributed using a balanced split-mouth design in the two maxillary quadrants, with one brand assigned to the anterior region and another to the posterior region on each side (Figure 1). At the midpoint of each phase (after 1.5 months), brackets were repositioned between anterior/posterior and contralateral regions to ensure equal regional exposure.

**Table 1:** Distribution of bracket groups according to material, composition, brand, manufacturer, color change (ΔE), NBS values, and clinical significance.

Crystal structure	Composition	Commercial name	Code	Manufacturer	ΔE (Mean ± SD)	NBS value (Mean ± SD)	Clinical significance
Ceramic	Monocrystalline	Gemini Clear Ceramic Brackets	Gemini	3M Unitek™, Monrovia, CA, USA	7.39 ± 2.38	6.80 ± 2.19	Extremely marked color change
Ceramic	Monocrystalline	Inspire ICE™	Ice	Ormco™, Orange, CA, USA	10.01 ± 3.65	9.20 ± 3.36	Extremely marked color change
Ceramic	Monocrystalline	Perfect Clear II	Hubit	Hubit™ Co. Ltd., Seoul, South Korea	9.74 ± 2.91	8.96 ± 2.68	Extremely marked color change
Ceramic	Monocrystalline	Radiance Plus™	Radiance	American Orthodontics™, Sheboygan, WI, USA	9.60 ± 3.94	8.84 ± 3.62	Extremely marked color change
Ceramic	Polycrystalline	Clarity™ Advanced	Clarity	3M™, Monrovia, CA, USA	11.17 ± 3.26	10.27 ± 2.99	Extremely marked color change
Ceramic	Polycrystalline	20/40™ Ceramic Brackets	20/40	American Orthodontics™, Sheboygan, WI, USA	6.98 ± 3.14	6.43 ± 2.89	Extremely marked color change
Ceramic	Polycrystalline	RESOLVE® C	Resolve	Dentsply Sirona™, New York, USA	8.82 ± 3.44	8.12 ± 3.17	Extremely marked color change
Ceramic	Polycrystalline	Chic Ceramic Bracket	Chic	GC Orthodontics, Alsip, IL, USA	10.07 ± 3.58	9.26 ± 3.30	Extremely marked color change

NBS: National Bureau of Standards; SD: standard deviation.

NBS = ΔE × 0.92. NBS criteria: 0.0–0.5 = trace; 0.5–1.5 = slight; 1.5–3.0 = noticeable; 3.0–6.0 = appreciable; 6.0–12.0 = extremely marked.

### Statistical analysis

Data normality was assessed using the Shapiro-Wilk test. As data were not normally distributed, nonparametric tests were applied: Mann–Whitney U test for between-group comparisons, Kruskal–Wallis test with post-hoc Dunn’s test for multiple group comparisons, and Wilcoxon signed-rank test for within-group comparisons. Effect sizes were calculated as rank-biserial correlation coefficients (*r*) for Mann–Whitney U tests (*r* < 0.10 negligible; 0.10–0.29 small; 0.30–0.49 medium; ≥ 0.50 large). For multiple post-hoc comparisons, Bonferroni correction was applied. The primary reporting format for non-normally distributed data was Median (interquartile range, IQR: Q1–Q3), with Mean ± SD provided as supplementary information. Statistical significance was set at *p* < 0.05. The lower and upper limits of the standard method error are presented in Table 2. Color differences were clinically interpreted using the NBS scale, in which values > 6 are classified as ‘extremely marked’ [20].

**Table 2:** Lower and upper limits of the standard method error.

Parameter	Std. method error	Lower limit	Upper limit
L	2.393	1.830	3.454
a	0.568	0.434	0.820
b	1.565	1.197	2.259
Tr	3.428	2.621	4.948

Method error calculated using the Dahlberg formula (ME = √(Σd²/2n)) based on 50 repeated measurements.

## Results

### Overview

All eight bracket brands exhibited measurable changes in color parameters (ΔL, Δa, Δb, ΔE) and light transmittance (ΔTr) following 3 months of intraoral exposure, with all brands reaching the ‘extremely marked’ discoloration category according to NBS classification criteria (NBS > 6) (Table 1).

**Figure 2:**
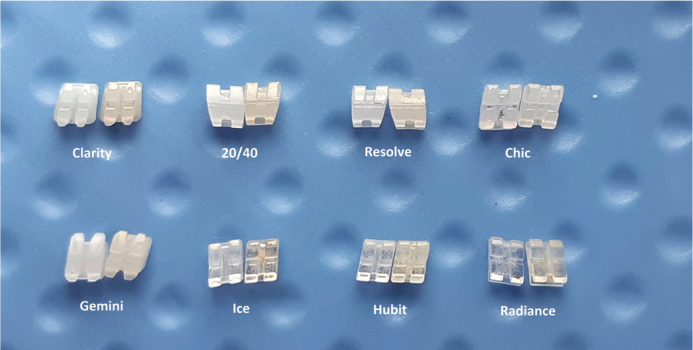
Before (left) and after (right) clinical use of the eight different branded brackets.

Per-brand analysis revealed brand-dependent variation in the magnitude of optical changes (Table 3). Among monocrystalline brackets, 3M™ Gemini Clear demonstrated the lowest color change, while Hubit™ Perfect Clear II showed the highest; Inspire Ice™ and AO™ Radiance Plus™ showed intermediate color changes. Regarding transmittance loss, AO™ Radiance Plus™ exhibited the greatest reduction, followed by Hubit™ Perfect Clear II. Among polycrystalline brackets, 3M™ Clarity™ Advanced demonstrated the highest color change, while AO™ 20/40 showed the lowest; Dentsply Sirona Resolve™ and GC™ Chic showed intermediate values. GC™ Chic exhibited the greatest transmittance loss among polycrystalline brackets, while 3M™ Clarity™ Advanced and Dentsply Sirona Resolve™ showed relatively smaller reductions. Visual comparison of bracket appearance before and after intraoral exposure is shown in Figure 2.

**Table 3:** Comparison of ΔL, Δa, Δb, ΔE, and ΔTr values of eight ceramic bracket brands (Kruskal–Wallis test).

Brand		ΔL	Δa	Δb	ΔE	ΔTr
**Gemini**	Median [IQR]	−3.85 [−6.50–−2.20]	−0.90 [−1.40–−0.10]	4.60 [3.30–5.90]	6.96 [5.73–8.83]	−5.00 [−15.00–−5.00]
	Mean ± SD	−4.41 ± 3.24	−0.81 ± 1.33	4.21 ± 3.23	7.39 ± 2.38	−9.40 ± 8.31
	Min / Max	−12.20 / 4.80	−4.50 / 2.30	−4.30 / 11.20	3.76 / 12.98	−35.00 / 0.00
**Ice**	Median [IQR]	−5.15 [−9.00–−1.90]	−0.50 [−1.20–0.00]	6.55 [5.60–8.30]	9.16 [7.49–11.50]	−5.00 [−10.00–0.00]
	Mean ± SD	−5.14 ± 5.44	−0.46 ± 0.91	7.04 ± 2.72	10.01 ± 3.65	−6.50 ± 6.80
	Min / Max	−19.00 / 7.20	−1.70 / 2.60	0.60 / 13.30	4.30 / 19.85	−25.00 / 0.00
**Hubit**	Median [IQR]	−3.00 [−4.70–0.40]	−0.35 [−1.20–1.00]	7.55 [6.70–9.70]	9.41 [7.90–11.70]	−10.00 [−15.00–−5.00]
	Mean ± SD	−2.53 ± 4.86	−0.07 ± 1.36	8.01 ± 2.80	9.74 ± 2.91	−11.10 ± 7.58
	Min / Max	−16.20 / 8.40	−1.80 / 5.10	−0.20 / 12.80	1.71 / 16.54	−30.00 / 0.00
**Radiance**	Median [IQR]	−4.10 [−7.70–−0.70]	−0.25 [−0.80–0.50]	6.65 [5.10–8.00]	9.05 [6.77–11.07]	−10.00 [−15.00–−5.00]
	Mean ± SD	−4.73 ± 5.49	−0.10 ± 1.22	6.98 ± 2.32	9.60 ± 3.94	−13.50 ± 17.82
	Min / Max	−19.70 / 5.70	−2.80 / 3.30	3.20 / 12.80	3.51 / 22.80	−125.00 / 0.00
**Clarity**	Median [IQR]	−6.50 [−9.10–−4.60]	0.80 [0.30–2.20]	8.85 [5.80–10.30]	11.22 [8.48–13.28]	−5.00 [−10.00–−5.00]
	Mean ± SD	−6.65 ± 3.94	1.29 ± 1.39	7.94 ± 3.03	11.17 ± 3.26	−7.20 ± 4.97
	Min / Max	−18.20 / 1.90	−0.60 / 4.60	0.70 / 14.40	5.10 / 21.17	−20.00 / 0.00
**20/40**	Median [IQR]	−2.05 [−5.90–1.30]	0.45 [−0.10–1.90]	3.25 [1.60–5.50]	6.67 [4.91–9.08]	−10.00 [−10.00–−5.00]
	Mean ± SD	−1.91 ± 5.40	0.86 ± 1.50	3.35 ± 3.24	6.98 ± 3.14	−8.90 ± 5.08
	Min / Max	−12.00 / 11.20	−3.70 / 4.10	−9.60 / 8.30	1.26 / 14.93	−20.00 / 0.00
**Resolve**	Median [IQR]	−5.85 [−8.50–−2.90]	−0.90 [−1.80–−0.40]	5.60 [3.20–7.60]	9.00 [6.19–11.25]	−5.00 [−10.00–0.00]
	Mean ± SD	−5.37 ± 4.46	−0.93 ± 1.35	5.38 ± 2.82	8.82 ± 3.44	−6.60 ± 6.01
	Min / Max	−14.20 / 8.20	−3.70 / 2.70	−0.60 / 11.10	1.24 / 16.31	−25.00 / 5.00
**Chic**	Median [IQR]	−4.75 [−8.60–2.30]	−0.15 [−0.50–0.40]	6.25 [4.90–7.50]	9.69 [7.34–11.91]	−10.00 [−15.00–−5.00]
	Mean ± SD	−4.05 ± 6.86	0.04 ± 0.97	4.92 ± 5.19	10.07 ± 3.58	−11.80 ± 8.85
	Min / Max	−19.00 / 10.20	−1.70 / 2.80	−10.20 / 11.80	3.87 / 19.60	−40.00 / 0.00
***p***		< 0.001	< 0.001	< 0.001	< 0.001	< 0.001

IQR: interquartile range; SD: standard deviation.

Kruskal–Wallis test. * *p* < 0.05; ** *p* < 0.001.

Δ values represent the difference between post-exposure and baseline measurements.

### Baseline and post-exposure optical parameters

Baseline and post-exposure values for lightness (L), red-green axis (a), blue-yellow axis (b), and light transmittance (Tr) are presented in Table 4. All eight bracket brands demonstrated statistically significant changes from baseline to post-exposure for L, b, and Tr (Wilcoxon signed-rank test, p < 0.05). Changes in the ‘a’ parameter were significant for five of the eight brands but not for Hubit, Radiance, or Chic. Significant between-brand differences were also observed at both baseline and post-exposure for all four parameters (Kruskal–Wallis test, *p* < 0.001).

**Table 4:** Comparison of baseline (L0, a0, b0, Tr0) and post-exposure (L1, a1, b1, Tr1) values of eight ceramic bracket brands (Wilcoxon signed-rank test).

Brand	L0 Mean ± SD	L1 Mean ± SD	*p*	a0 Mean ± SD	a1 Mean ± SD	*p*	b0 Mean ± SD	b1 Mean ± SD	*p*	Tr0 Mean ± SD	Tr1 Mean ± SD	*p*
Gemini	96.16 ± 2.94	91.75 ± 3.10	< 0.001**	2.03 ± 1.15	1.22 ± 1.13	< 0.001**	2.69 ± 1.41	6.90 ± 2.68	< 0.001**	231.90 ± 12.53	222.50 ± 13.33	< 0.001**
Ice	98.89 ± 2.06	93.76 ± 4.66	< 0.001**	1.16 ± 0.49	0.70 ± 0.92	< 0.001**	2.16 ± 2.35	9.20 ± 2.75	< 0.001**	227.80 ± 13.10	221.30 ± 13.92	< 0.001**
Hubit	97.79 ± 3.32	95.26 ± 3.33	0.001**	1.17 ± 1.03	1.10 ± 1.04	0.362 (ns)	2.18 ± 1.16	10.19 ± 2.37	< 0.001**	253.20 ± 11.28	242.00 ± 10.83	< 0.001**
Radiance	98.52 ± 2.04	93.79 ± 5.51	< 0.001**	1.42 ± 0.49	1.32 ± 1.07	0.254 (ns)	1.71 ± 0.40	8.69 ± 2.27	< 0.001**	264.10 ± 4.81	250.60 ± 17.52	< 0.001**
Clarity	97.16 ± 2.26	90.51 ± 3.13	< 0.001**	−0.31 ± 0.70	0.98 ± 0.93	< 0.001**	2.13 ± 1.38	10.08 ± 2.63	< 0.001**	221.60 ± 7.52	214.40 ± 9.51	< 0.001**
20/40	92.92 ± 4.59	91.01 ± 3.23	0.012*	0.50 ± 1.08	1.36 ± 0.84	0.001**	3.91 ± 1.93	7.26 ± 2.70	< 0.001**	228.40 ± 7.32	219.50 ± 9.22	< 0.001**
Resolve	96.57 ± 3.63	91.20 ± 3.50	< 0.001**	2.44 ± 1.30	1.52 ± 0.84	< 0.001**	2.85 ± 1.47	8.22 ± 2.49	< 0.001**	224.10 ± 5.02	217.50 ± 6.80	< 0.001**
Chic	93.21 ± 5.34	89.16 ± 4.74	< 0.001**	1.30 ± 0.44	1.34 ± 0.77	0.526 (ns)	2.70 ± 2.94	7.61 ± 2.96	< 0.001**	212.50 ± 11.44	200.70 ± 15.94	< 0.001**
*p* (between brands)	< 0.001**	< 0.001**	-	< 0.001**	< 0.001**	-	< 0.001**	< 0.001**	-	< 0.001**	< 0.001**	-

ns: non-significant; SD: standard deviation.

Wilcoxon signed-rank test (within-group comparisons). Kruskal–Wallis test (between-group comparisons).

* *p* < 0.05;

** *p* < 0.001.

### Intragroup comparisons within crystalline structure groups

Post-hoc pairwise comparisons within monocrystalline and polycrystalline groups revealed significant brand-dependent differences in the magnitude of optical changes (Table 5). Within the monocrystalline group, 3M™ Gemini Clear demonstrated significantly lower ΔE values than the other three monocrystalline brands (*p* < 0.05). Within the polycrystalline group, AO™ 20/40 demonstrated significantly lower ΔE values than the other three polycrystalline brands (*p* < 0.05).

**Table 5a:** Intragroup post-hoc comparisons of ΔL, Δa, Δb, ΔE, and ΔTr within polycrystalline and monocrystalline bracket groups.

Polycrystalline Group							
Parameter	Clarity–20/40	Clarity–Resolve	Clarity–Chic	20/40–Resolve	20/40–Chic	Chic–Resolve	Overall *p*
ΔL	< 0.001**	ns	ns	0.011*	ns	ns	< 0.001**
Δa	ns	< 0.001**	< 0.001**	< 0.001**	0.019*	0.012*	< 0.001**
Δb	< 0.001**	0.001**	0.006*	0.041*	0.005*	ns	< 0.001**
ΔE	< 0.001**	0.013*	ns	0.044*	< 0.001**	ns	< 0.001**
ΔTr	ns	ns	0.045*	ns	ns	0.005*	< 0.001**

**Table 5b:** Intragroup post-hoc comparisons of ΔL, Δa, Δb, ΔE, and ΔTr within polycrystalline and monocrystalline bracket groups.

Monocrystalline Group							
Parameter	Gemini–Radiance	Gemini–Ice	Gemini–Hubit	Hubit–Ice	Radiance–Ice	Hubit–Radiance	Overall *p*
ΔL	ns	ns	ns	0.045*	ns	ns	0.044*
Δa	ns	ns	0.018*	ns	ns	ns	0.014*
Δb	< 0.001**	< 0.001**	< 0.001**	ns	ns	ns	< 0.001**
ΔE	0.001**	< 0.001**	0.008*	ns	ns	ns	< 0.001**
ΔTr	ns	ns	ns	0.008*	0.002*	ns	< 0.001**

ns: non-significant.

Post-hoc analysis with Bonferroni correction applied for multiple comparisons.

* *p* < 0.05;

** *p* < 0.001.

Δ values represent the difference between post-exposure and baseline measurements.

### Intergroup comparison of Δ parameters

Comparison of ΔL, Δa, Δb, ΔE, and ΔTr between monocrystalline and polycrystalline bracket groups revealed statistically significant differences for Δa and Δb only (Table 6). The median Δa value was significantly lower in the monocrystalline group, with a medium effect size, while the median Δb value was significantly higher in the monocrystalline group, with a small effect size. No statistically significant intergroup differences were found for ΔL, ΔE, or ΔTr.

**Table 6:** Intergroup comparison of ΔL, Δa, Δb, ΔE, and ΔTr between monocrystalline and polycrystalline bracket groups (Mann–Whitney U test).

Parameter	Monocrystalline Mean ± SD	Monocrystalline Median [IQR]	Polycrystalline Mean ± SD	Polycrystalline Median [IQR]	*p*	Effect size (*r*)	Interpretation
ΔL	−4.20 ± 4.91	−3.85 [−7.70 to −0.70]	−4.49 ± 5.53	−4.75 [−8.60 to 1.30]	0.178	0.077	Negligible
Δa	−0.36 ± 1.25	−0.50 [−1.20 to 0.00]	0.32 ± 1.56	0.45 [−0.10 to 1.90]	< 0.001**	0.301	Medium
Δb	6.56 ± 3.11	6.50 [3.30 to 8.30]	5.40 ± 4.02	6.00 [3.25 to 8.85]	0.011*	0.147	Small
ΔE	9.18 ± 3.42	9.05 [6.77 to 11.50]	9.26 ± 3.68	9.69 [7.34 to 13.28]	0.570	0.033	Negligible
ΔTr	−10.12 ± 11.28	−10.00 [−15.00 to −5.00]	−8.62 ± 6.69	−10.00 [−10.00 to −5.00]	0.362	0.051	Negligible

IQR: interquartile range; SD: standard deviation.

Mann–Whitney U test.

* *p* < 0.05;

** *p* < 0.001.

Effect size *r* (rank-biserial correlation): negligible < 0.10; small 0.10–0.29; medium 0.30–0.49; large ≥ 0.50.

### Intergroup comparison of baseline and post-exposure parameters

Comparison of baseline and post-exposure L, a, b, and Tr values between monocrystalline and polycrystalline groups revealed significant intergroup differences in L0, a0, b0, and Tr0 (Table 7). Monocrystalline brackets showed significantly higher L0, a0, and Tr0 values, but significantly lower b0 values, than polycrystalline brackets. At the post-exposure timepoint, the intergroup differences in L1 and Tr1 persisted in the same direction, the difference in b1 was no longer significant, and the direction of the difference in a1 was reversed, with polycrystalline brackets showing higher values than monocrystalline brackets.

**Table 7:** Intergroup comparison of baseline and post-exposure L, a, b, and Tr values between monocrystalline and polycrystalline bracket groups (Mann–Whitney U test).

Parameter	Monocrystalline Mean ± SD	Monocrystalline Min / Max	Within-group *p* (Wilcoxon)	Polycrystalline Mean ± SD	Polycrystalline Min / Max	Within-group *p* (Wilcoxon)	Between-group *p* (Mann–Whitney U)
L0	97.84 ± 2.83	85.2 / 102.0	< 0.001**	94.97 ± 4.52	80.5 / 100.7	< 0.001**	< 0.001**
L1	93.64 ± 4.41	77.4 / 100.0	< 0.001**	90.47 ± 3.76	78.8 / 100.0	< 0.001**	< 0.001**
a0	1.44 ± 0.91	−4.2 / 5.7	< 0.001**	0.98 ± 1.38	−1.7 / 5.1	0.020*	0.020*
a1	1.08 ± 1.06	−0.4 / 4.8	< 0.001**	1.30 ± 0.86	−1.0 / 4.0	0.020*	0.020*
b0	2.19 ± 1.53	0.8 / 13.4	< 0.001**	2.90 ± 2.11	0.8 / 15.2	< 0.001**	< 0.001**
b1	8.75 ± 2.78	1.1 / 15.1	< 0.001**	8.29 ± 2.89	1.2 / 16.5	< 0.001**	0.184 (ns)
Tr0	244.25 ± 18.53	195.0 / 285.0	< 0.001**	221.65 ± 9.98	190.0 / 240.0	< 0.001**	< 0.001**
Tr1	234.10 ± 18.84	140.0 / 275.0	< 0.001**	213.03 ± 13.09	160.0 / 240.0	< 0.001**	< 0.001**

ns: non-significant. SD: standard deviation.

Mann–Whitney U test (between-group comparisons). Wilcoxon signed-rank test (within-group comparisons).

*p < 0.05;

** *p* < 0.001.

### Correlation between ΔE and ΔTr

A weak but statistically significant negative correlation was observed between ΔE and ΔTr in both bracket groups (Table 8). Although this correlation reached statistical significance due to the large sample size (*n* = 200 per group), the effect size is small, and the clinical relevance of this association is limited. It should not be interpreted as evidence of a clinically meaningful relationship between color change and transmittance loss on an individual patient basis.

**Table 8:** Spearman correlation between color change (ΔE) and light transmittance change (ΔTr) in monocrystalline and polycrystalline bracket groups.

Group	Parameter	*r*	*p*	Effect size interpretation	Clinical relevance
Monocrystalline	ΔE vs. ΔTr	−0.173	0.014*	Weak (negligible–small)	Limited – statistically significant due to large n; not clinically meaningful at individual level
Polycrystalline	ΔE vs. ΔTr	−0.161	0.023*	Weak (negligible–small)	Limited – statistically significant due to large n; not clinically meaningful at individual level

Spearman’s rank correlation coefficient.

* *p* < 0.05.

Effect size interpretation: |*r*| < 0.10 = negligible; 0.10–0.29 = small; 0.30–0.49 = medium; ≥ 0.50 = large.

### Comparison of clinical exposure versus control groups

Brackets exposed to the oral cavity exhibited significantly higher ΔE values and greater reductions in ΔTr than control groups stored in artificial saliva, for all eight bracket brands (Kruskal–Wallis test, *p* < 0.05; Table 9), underscoring the limitations of in vitro simulations in replicating the complexity of the oral environment.

**Table 9:** Comparison of ΔE and ΔTr values between clinical exposure groups and control groups stored in artificial saliva (Kruskal–Wallis test).

Crystal structure	Brand	Parameter	Experimental group Mean ± SD	Experimental group Min / Max	Control group Mean ± SD	Control group Min / Max	*p*
Monocrystalline	Gemini	ΔE	7.39 ± 2.38	3.76 / 12.98	1.50 ± 0.90	0.80 / 3.00	< 0.001**
Monocrystalline	Gemini	ΔTr	−9.40 ± 8.31	−35.00 / 0.00	0.00 ± 0.00	0.00 / 0.00	0.002*
Monocrystalline	Ice	ΔE	10.01 ± 3.65	4.30 / 19.85	1.60 ± 0.70	0.60 / 2.30	< 0.001**
Monocrystalline	Ice	ΔTr	−6.50 ± 6.80	−25.00 / 0.00	0.00 ± 0.00	0.00 / 0.00	0.011*
Monocrystalline	Hubit	ΔE	9.74 ± 2.91	1.71 / 16.54	1.80 ± 3.40	0.00 / 7.70	0.001**
Monocrystalline	Hubit	ΔTr	−11.10 ± 7.58	−30.00 / 0.00	0.00 ± 0.00	0.00 / 0.00	0.001**
Monocrystalline	Radiance	ΔE	9.60 ± 3.94	3.51 / 22.80	1.80 ± 0.80	0.40 / 2.70	< 0.001**
Monocrystalline	Radiance	ΔTr	−13.50 ± 17.82	−125.00 / 0.00	−1.00 ± 2.20	−5.00 / 0.00	0.003*
Polycrystalline	Clarity	ΔE	11.17 ± 3.26	5.10 / 21.17	0.60 ± 0.40	0.00 / 1.10	< 0.001**
Polycrystalline	Clarity	ΔTr	−7.20 ± 4.97	−20.00 / 0.00	0.00 ± 0.00	0.00 / 0.00	0.002*
Polycrystalline	20/40	ΔE	6.98 ± 3.14	1.26 / 14.93	0.60 ± 0.20	0.30 / 0.70	< 0.001**
Polycrystalline	20/40	ΔTr	−8.90 ± 5.08	−20.00 / 0.00	0.00 ± 0.00	0.00 / 0.00	0.001**
Polycrystalline	Resolve	ΔE	8.82 ± 3.44	1.24 / 16.31	0.40 ± 0.20	0.20 / 0.70	< 0.001**
Polycrystalline	Resolve	ΔTr	−6.60 ± 6.01	−25.00 / 5.00	0.00 ± 0.00	0.00 / 0.00	0.006*
Polycrystalline	Chic	ΔE	10.07 ± 3.58	3.87 / 19.60	3.70 ± 1.90	2.30 / 7.00	0.001**
Polycrystalline	Chic	ΔTr	−11.80 ± 8.85	−40.00 / 0.00	0.00 ± 0.00	0.00 / 0.00	0.001**

SD: standard deviation.

Kruskal–Wallis test.

* *p* < 0.05;

** *p* < 0.001.

Δ values represent the difference between post-exposure and baseline measurements.

## Discussion

### Extrinsic factors and color change

This clinical investigation assessed the in vivo color stability and light transmittance changes of monocrystalline and polycrystalline ceramic brackets following 3 months of intraoral exposure. The results revealed significant alterations in color parameters (ΔE, Δa, Δb, ΔL) and reductions in light transmittance (ΔTr) for all tested brands, indicating that the optical properties of aesthetic ceramic brackets are vulnerable to intraoral aging. The combined optical deterioration observed in this study is consistent with the concept of intraoral aging described by Eliades and Bourauel [21], whereby exposure to the oral environment can induce surface and compositional alterations in orthodontic materials that are not reproduced in artificial media. Brackets exposed to the oral cavity exhibited significantly higher ΔE values and greater reductions in ΔTr compared to control groups stored in artificial saliva, underscoring the limitations of in vitro simulations in replicating the complexity of the oral environment.

In the present investigation, all tested brackets demonstrated ‘extremely marked’ color changes according to the NBS classification (NBS: 6.43–10.27 units) after 3 months of clinical exposure, with a significant increase in yellowness (b*) and corresponding darkening. These results align with those of Shibani et al., who observed significant color shifts in ceramic brackets among adolescent patients, particularly when exposed to staining beverages [18]. Similarly, Guignone et al. reported that ceramic brackets immersed in commonly consumed beverages exhibited progressive color alterations [9]. Beyond dietary factors, tobacco smoke, ethnic food pigments, and surface roughness have also been shown to contribute to bracket discoloration [8, 17, 22]. AlBadr and Talic [23] further emphasized that reflected color coordinates, opalescence, and translucency are significantly influenced by the material’s microstructure.

### Material properties and crystalline structure

Regarding the first study hypothesis, the null hypothesis that short-term intraoral exposure would not produce significant changes in color stability or light transmittance was rejected. All eight bracket brands demonstrated statistically significant changes in both ΔE and ΔTr after 3 months of intraoral exposure, confirming that intraoral aging has a measurable and clinically relevant impact on the optical properties of ceramic brackets. Eliades et al. [14] and Mohamed et al. [24] demonstrated that structure and composition affect direct light transmission, with polycrystalline brackets showing higher light absorption and less translucency. The simultaneous evaluation of light transmittance in this study is consistent with the approach of Lopes Filho et al. [25], who assessed the influence of esthetic brackets’ optical properties on visual perception. Color and fluorescence changes have similarly been documented in other esthetic orthodontic components, such as archwires [26].

Regarding the second study hypothesis, our findings indicate that crystalline structure partially influences optical performance. Specifically, crystalline structure significantly influenced baseline and final transmittance values (Tr0, Tr1) and baseline chromatic parameters, with monocrystalline brackets showing higher L0, L1, a0, Tr0, and Tr1 values. However, crystalline structure did not determine the overall magnitude of color change (ΔE) or transmittance loss (ΔTr) between the two groups (no statistically significant difference). Therefore, the second hypothesis is partially rejected: crystalline structure influences baseline optical properties but not the overall magnitude of optical degradation. Effect size analysis revealed that while the overall magnitude of color change (ΔE) did not differ significantly between monocrystalline and polycrystalline brackets, the chromatic axis shift (Δa) showed a medium effect (statistically significant), indicating that crystalline structure systematically influenced the direction of color change rather than its magnitude. Monocrystalline brackets demonstrated a more pronounced shift toward negative a-axis values (green direction), while polycrystalline brackets showed a shift toward positive a-axis values (red/yellow direction). Additionally, a small but significant effect was observed for Δb (statistically significant), with monocrystalline brackets exhibiting slightly higher yellow-axis shift.

### Clinical implications

The findings of this study provide short-term clinical evidence that the optical properties of ceramic brackets are susceptible to intraoral degradation within 3 months. Recent developments in customized ceramic brackets aim to improve aesthetic performance through individualized color and translucency adjustments [27]. Given the 3-month observation period of the present study, it would be premature to draw definitive long-term clinical conclusions. Longer-term prospective studies are needed to determine whether the optical changes observed here continue to progress, plateau, or accelerate over the full duration of orthodontic treatment. Within the limitations of the present study, clinicians may consider discussing the potential for aesthetic changes with patients, particularly those with high aesthetic demands, while acknowledging that individual outcomes may vary substantially based on dietary habits and oral hygiene practices. As newer esthetic orthodontic bracket systems, including 3D-printed brackets, continue to emerge, their long-term optical stability under intraoral conditions, including both color and translucency, warrants further clinical investigation [28, 29].

## Limitations

The present study has several limitations that should be considered when interpreting the findings:

1. **Blinding:** Although allocation to the first phase was randomized, blinding of participants and assessors was not feasible given the nature of the intervention, which may introduce performance or detection bias.
2. **Absence of adhesive bonding (structural limitation):** No adhesive composite was bonded under the brackets to prevent composite-induced discoloration. This represents a structural methodological limitation, as brackets are invariably bonded in clinical practice. The absence of adhesive bonding may have underestimated actual optical changes and excluded the contribution of adhesive materials to overall aesthetic outcomes.
3. **Transmittance measurement:** Light transmittance was assessed using an LED curing unit and a radiometer, which measures transmitted light intensity rather than absolute spectral optical transmittance. The absence of spectral analysis and true transmission spectrophotometry limits comparability with studies employing spectrofluorometer-based methods [24].
4. **Multiple comparisons:** Bonferroni correction was applied for post-hoc comparisons; however, the risk of Type I error cannot be entirely eliminated given the number of intergroup analyses.
5. **Dietary control:** Dietary intake was not objectively controlled beyond standardized dietary advice and recall questionnaires, which may have introduced intra-group variability.
6. **Short follow-up:** The 3-month observation period represents short-term clinical aging only and may not fully reflect long-term intraoral behavior.

## Conclusions

After 3 months of intraoral exposure, all evaluated ceramic brackets demonstrated statistically significant, brand-dependent changes in color (ΔE) and light transmittance (ΔTr). All brands exhibited ‘extremely marked’ discoloration according to NBS criteria (NBS > 6). A weak negative correlation was identified between color degradation and transmittance reduction, though the clinical significance of this association is limited.

Crystalline structure (monocrystalline vs. polycrystalline) influenced baseline optical properties – including initial lightness and transmittance values – but did not significantly determine the overall magnitude of color change (ΔE) or transmittance loss (ΔTr). However, crystalline structure did influence the direction of chromatic shift, with monocrystalline brackets showing a medium-effect difference in Δa and a small-effect difference in Δb. Total optical stability appeared to be primarily influenced by brand-specific manufacturing characteristics rather than crystalline structure alone. Within the constraints of the 3-month follow-up period and the absence of adhesive bonding, these findings suggest that clinicians should consider brand-specific optical performance when selecting ceramic brackets for patients with high aesthetic demands.

## Data Availability

The data that support the findings of this study are available from the corresponding author upon reasonable request.

## References

[1] Faltermeier A, Behr M, Müssig D. In vitro colour stability of aesthetic brackets. Eur J Orthod. 2007;29(4):354–8. 10.1093/ejo/cjm020 17702794

[2] Jena A, Duggal R, Mehrotra A. Physical properties and clinical characteristics of ceramic brackets: a comprehensive review. Trends Biomater Artif Organs. 2007;20(1):1–10.

[3] Birnie D. Ceramic brackets. Br J Orthod. 1990;17(1):71–4. 10.1179/bjo.17.1.71 2178681

[4] Trakyalı G. Evaluation of orthodontic adhesive color reflection degrees of transparent and translucent ceramic brackets. Atatürk Univ Dis Hekim Fak Derg. 2020;40(2):123–30.

[5] Russell JS. Aesthetic orthodontic brackets. J Orthod. 2005;32(2): 146–63. 10.1179/146531205225021024 15994990

[6] Yu B, Lee YK. Aesthetic colour performance of plastic and ceramic brackets: an in vitro study. J Orthod. 2011;38(3):167–74. 10.1179/14653121141434 21875990

[7] Silva DL, Mattos CT, de Araújo MVA, Ruellas ACO. Color stability of ceramic brackets immersed in artificial saliva and a potentially staining health drink solution. Dent Press J Orthod. 2023;28(1):e2023001. 10.1590/2177-6709.28.1.e2023001

[8] Quadros AR, Ferretti MA, Aguiar FHB, Basting RT. Color change and surface degradation of esthetic brackets after exposure to cigarette smoke. Acta Odontol Latindom. 2025;38(1):39–48. 10.54589/aol.38/1/39 PMC12317772 40741806

[9] Guignone BC, Silva LK, Soares RV, Akaki E, Goiato MC, Pithon MM. Color stability of ceramic brackets immersed in potentially staining solutions. Dent Press J Orthod. 2015;20(4):32–8. 10.1590/2176-9451.20.4.032-038.oar PMC4593527 26352842

[10] Filho HL, Maia LH, Araújo MV, Eliades T, Ruellas AC. Colour stability of aesthetic brackets: ceramic and plastic. Aust Orthod J. 2013;29(1): 13–20. 10.2478/aoj-2013-0003 23785933

[11] Khashayar G, Bain PA, Salari S, Dozic A, Kleverlaan CJ, Feilzer AJ. Perceptibility and acceptability thresholds for colour differences in dentistry. J Dent. 2014;42(6):637–44. 10.1016/j.jdent.2013.11.017 24334221

[12] Heffernan MJ, Aquilino SA, Diaz-Arnold AM, Haselton DR, Stanford CM, Vargas MA. Relative translucency of six all-ceramic systems. Part I: core materials. J Prosthet Dent. 2002;88(1):4–9. 10.1067/mpr.2002.126794 12239472

[13] Karamouzos A, Athanasiou AE, Papadopoulos MA. Clinical characteristics and properties of ceramic brackets: a comprehensive review. Am J Orthod Dentofacial Orthop. 1997;112(1):34–40. 10.1016/S0889-5406(97)70271-3 9228839

[14] Eliades T, Johnston WM, Eliades G. Direct light transmittance through ceramic brackets. Am J Orthod Dentofacial Orthop. 1995;107(1): 11–9. 10.1016/s0889-5406(95)70152-4 7817957

[15] de Oliveira CB, Maia LGM, Santos-Pinto A, Gandini LG Jr. In vitro study of color stability of polycrystalline and monocrystalline ceramic brackets. Dent Press J Orthod. 2014;19(4):114–21. 10.1590/2176-9451.19.4.114-121.oar PMC4296644 25279530

[16] Šimunović L, Blagec T, Vrankić A, Meštrović S. Color stability of orthodontic ceramic brackets and adhesives in potentially staining beverages: in vitro study. Dent J (Basel). 2022;10(7):115. 10.3390/dj10070115 35877389 PMC9351677

[17] Pithon MM, Baião FS, Sant’Anna LI, Paranhos LR, Coqueiro RS. Effect of different concentrations of chlorhexidine-based mouthwashes on color stability of orthodontic brackets: an in vitro study. J Appl Oral Sci. 2014;22(5):433–9. 10.1590/1678-775720130619

[18] Shibani AM, de Oliveira CB, Gandini LG Jr, Maia LGM. Colour change of ceramic brackets with the use of coloured beverages in adolescent patients: a randomized clinical trial. Prog Orthod. 2020;21(1):45. 10.1186/s40510-020-00329-2 32147329

[19] Öz AA, Öz AZ, Canlı E, Çelebi F. Comparison of color stability of different esthetic brackets. Ondokuz Mayis Univ Dis Hekim Fak Derg. 2012;13(1):7–12.

[20] Kuehni RG, Marcus RT. An experiment in visual scaling of small color differences. Color Res Appl. 1979;4(2):83–91. 10.1002/col.5080040206

[21] Eliades T, Bourauel C. Intraoral aging of orthodontic materials: the picture we miss and its clinical relevance. Am J Orthod Dentofacial Orthop. 2005;127(4):403–12. 10.1016/j.ajodo.2004.09.015 15821684

[22] Borges L, Castro ACR, Elias CN, Souza MMG. Effect of cigarette smoke on aesthetic brackets: an in vitro study. Dent Press J Orthod. 2022;27(4):e2220365. 10.1590/2177-6709.27.4.e2220365.oar PMC9439572 36074431

[23] AlBadr AH, Talic NF. The optical translucency, opalescence, and reflectance of different as-received ceramic brackets: a spectrophotometer study. J Esthet Restor Dent. 2023;35(1):125–36. 10.1111/jerd.12983

[24] Mohamed JP, Kommi PB, Kumar MS. Evaluating the type of light transmittance in mono crystalline, poly crystalline and sapphire brackets: an in vitro spectrofluorometer study. J Clin Diagn Res. 2016;10(8):ZC18–21. 10.7860/JCDR/2016/18599.8230 PMC5028516 27656556

[25] Lopes Filho HL, Maia LEG, Araújo MVA, Ruellas ACO. Influence of optical properties of esthetic brackets on visual perception. Am J Orthod Dentofacial Orthop. 2012;141(4):460–7. 10.1016/j.ajodo.2011.10.026 22464528

[26] da Silva DL, Mattos CT, de Araújo MVA, Ruellas ACO. Color stability and fluorescence of different orthodontic esthetic archwires. Angle Orthod. 2013;83(1):127–32. 10.2319/121311-764.1 22591261 PMC8805526

[27] Yang L, Yin G, Liao X, Yin X, Ye N. A novel customized ceramic bracket for esthetic orthodontics: in vitro study. Prog Orthod. 2019;20(1):39. 10.1186/s40510-019-0292-y 31608421 PMC6790353

[28] Wallach R, English JD, Moon A, Brock RA, Paravina RD, Kasper FK. Colour stability of 3D-printed orthodontic brackets using filled resins. Orthod Craniofac Res. 2023;26(Suppl 1):180–7. 10.1111/ocr.12665 37089069

[29] Brucculeri L, Pellitteri F, Falconi V, Palone M, Lombardo L. Colour stability between in-house 3D-printed resin brackets and conventionally aesthetic brackets: an in vitro study. Appl Sci. 2024;14(13):5753. 10.3390/app14135753

